# Comparison of Proton Acceptor and Proton Donor Properties of H_2_O and H_2_O_2_ in Organic Crystals of Drug-like Compounds: Peroxosolvates vs. Crystallohydrates

**DOI:** 10.3390/molecules27030717

**Published:** 2022-01-22

**Authors:** Mikhail V. Vener, Andrei V. Churakov, Alexander P. Voronin, Olga D. Parashchuk, Sergei V. Artobolevskii, Oleg A. Alatortsev, Denis E. Makhrov, Alexander G. Medvedev, Aleksander Filarowski

**Affiliations:** 1 Kurnakov Institute of General and Inorganic Chemistry, Russian Academy of Sciences, Leninskii Prosp. 31, 119991 Moscow, Russia; churakov@igic.ras.ru (A.V.C.); mag@igic.ras.ru (A.G.M.); 2 G.A. Krestov Institute of Solution Chemistry RAS, 153045 Ivanovo, Russia; apv@isc-ras.ru; 3 Faculty of Physics, Lomonosov Moscow State University, 119991 Moscow, Russia; olga_par@physics.msu.ru; 4 Faculty of Natural Science, Mendeleev University of Chemical Technology, Miusskaya Square 9, 125047 Moscow, Russia; Artobolevskiy_Sergey_Vladimirovich@muctr.ru (S.V.A.); Olegalatorcev@muctr.ru (O.A.A.); Denis_Makhrov@muctr.ru (D.E.M.); 5 Faculty of Chemistry, University of Wrocław 14 F. Joliot-Curie Str., 50-383 Wrocław, Poland

**Keywords:** crystal packing, periodic DFT computations, bifurcate hydrogen bonds, low-frequency Raman spectroscopy, hydrogen bond enthalpy

## Abstract

Two new peroxosolvates of drug-like compounds were synthesized and studied by a combination of X-ray crystallographic, Raman spectroscopic methods, and periodic DFT computations. The enthalpies of H-bonds formed by hydrogen peroxide (H_2_O_2_) as a donor and an acceptor of protons were compared with the enthalpies of analogous H-bonds formed by water (H_2_O) in isomorphic (isostructural) hydrates. The enthalpies of H-bonds formed by H_2_O_2_ as a proton donor turned out to be higher than the values of the corresponding H-bonds formed by H_2_O. In the case of H_2_O_2_ as a proton acceptor in H-bonds, the ratio appeared reversed. The neutral O∙∙∙H-O/O∙∙∙H-N bonds formed by the lone electron pair of the oxygen atom of water were the strongest H-bonds in the considered crystals. In the paper, it was found out that the low-frequency Raman spectra of isomorphous crystalline hydrate and peroxosolvate of N-(5-Nitro-2-furfurylidene)-1-aminohydantoin are similar. As for the isostructural hydrate and peroxosolvate of the salt of protonated 2-amino-nicotinic acid and maleic acid monoanion, the Raman spectra are different.

## 1. Introduction

In the last decade, the development of drug-like cocrystals became one of the topical issues in pharmaceutical chemistry [1,2,3], owing to a possible synergetic effect of their components [4]. The hydrogen peroxide crystalline complexes look very promising since H_2_O_2_ demonstrates a wide spectrum of antimicrobial activity [5]. Recently, the peroxosolvate of the antifungal drug “miconazole” was synthesized and structurally characterized [6]. However, upon storage, peroxosolvates get decomposed with hydrogen peroxide leaking away, and, therefore, they are not applicable for medical treatment. The formation of stoichiometric H_2_O_2_ adduct with no loss of oxidizing ability upon long-term storage is one of key challenges [7]. It is well-known that the stability of peroxosolvates is governed by the strength and amount of hydrogen bonds formed by peroxide molecules [8,9] and the topology of H-bonded networks within their crystals [10,11].

The conventional hydrogen bonds (H-bonds) are the main type of intermolecular interaction in crystallohydrates and crystalline peroxosolvates. Water and hydrogen peroxide are able to form a different number of H-bonds in multicomponent organic crystals. H_2_O usually forms three H-bonds: either two bonds as a proton donor and one bond as a proton acceptor or vice versa [11,12,13]. H_2_O_2_ always forms two conventional H-bonds as a proton donor and may form up to four H-bonds as a proton acceptor [14]. The proton-donor atoms are usually formed with aromatic nitrogen, N_AR_ [9], and the ^−^O-N^+^_AR_ [10], ^−^O_2_CR [15], O=CR_2_ [16] groups. Such H-bonds are quasi-linear (the O-H∙∙∙O/O-H∙∙∙N angle is greater than 160 degrees) and are of moderate strength [9,15,16]. H_2_O_2_ forms three or four H-bonds as a proton acceptor in a few crystals [9]. These bonds are formed between lone electron pairs of the oxygen atom of H_2_O_2_ and π-conjugated amino groups of organic coformers [9]. They are characterized by an almost linear O∙∙∙H-N fragment and are relatively weak [9,16]. The H_2_O_2_ molecule forms one or two H-bonds as a proton acceptor in about half of the peroxosolvates [14]. A significant number of these crystals contain zwitterions [17,18,19]. The oxygen of the H_2_O_2_ molecule and the ^+^H_3_N- group of the amino acid zwitterion often form nonlinear H-bonds (the O∙∙∙H-N^+^ angle is less than 140 degrees), Figure S1.

The distance between the A and B atoms (*R*(A∙∙∙B)) of the A-H∙∙∙B fragment, where A and B are O, N, F and others, plays a crucial role in geometry, dynamics, and in the energy of the H-bond network [20,21]. The different properties of H-bonds are determined by the *R*(A∙∙∙B) distance, in particular, the energy/enthalpy of H-bond (*E*_HB_/Δ*H_HB_*). The proposed empirical schemes made it possible to estimate the *E*_HB_ values from *R*(O∙∙∙O) [22,23]. These approaches are limited to the O-H∙∙∙O fragment in the solid state. More universal approaches use the frequency shifts of the O-H stretching vibrations [24], the H∙∙∙O distance [25,26] and the electron density at the bond critical point [27]. The pros and cons of various schemes for *E*_HB_/Δ*H_HB_* estimating are given elsewhere [28]. In the present study, the Δ*H_HB_* values were evaluated using the Rozenberg equation [25]:

–Δ*H_HB_* [kJ mol^−1^] = 0.134·*R*(H∙∙∙O) ^−3.05^, (1)

where the *R*(H∙∙∙O) is the H∙∙∙O distance (nm). The empirical correlation (1) gives the Δ*H_HB_* values of intermolecular H-bonds in molecular crystals in the range of 10−80 kJ/mol with the accuracy around several kJ/mol [25]. The Equation (1) is a powerful “toolbox” for studying crystals consisting of H-bonds of different strengths and types, including ionic or charged fragments [25,26]. The main limitation of equation (1) is the accuracy of experimental measurements of the position of hydrogen in H-bridge. Therefore, the use of the neutron diffraction method is necessary. Nevertheless, the number of crystals with H-bonds studied by this method is very limited [29]. In the present study, the exact values of the H∙∙∙O distances were computed using the periodic DFT methods [30,31].

The energy/enthalpy of H-bonds in peroxosolvates composed by H_2_O_2_ as a proton donor is usually greater than the corresponding *E*_HB_/Δ*H_HB_* of H-bonds formed by the H_2_O molecule [15,32]. A systematic comparison of the *E*_HB_/Δ*H_HB_* values of H-bonds formed by H_2_O and H_2_O_2_ as proton acceptors has not been performed so far. In paper [13] it is believed that the energies of moderately strong H-bonds of complexes formed by the H_2_O molecule as a proton donor or a proton acceptor are approximately equal. As for multicomponent organic crystals, this equality may not be observed, since the energies of the H-bonds formed by H_2_O and H_2_O_2_ as proton acceptors are determined by the nature of the organic molecule. Indeed, the *E*_HB_/Δ*H_HB_* values of the H-bond built up by the H_2_O molecule as a proton acceptor with a diglycine cyclic dipeptide are greater than the values of the corresponding H-bond formed by H_2_O_2_ [32]. Obviously, an accurate comparison of the energy of the H-bond formed by H_2_O and H_2_O_2_ as acceptors of protons in the solid state involves the study of multicomponent organic crystals that form isomorphic (isostructural) hydrates and peroxosolvates.

To compare the proton acceptor and proton donor properties of H_2_O and H_2_O_2_ in multicomponent organic crystals, the following issues were consistently resolved in this work.

**Scheme 1:**
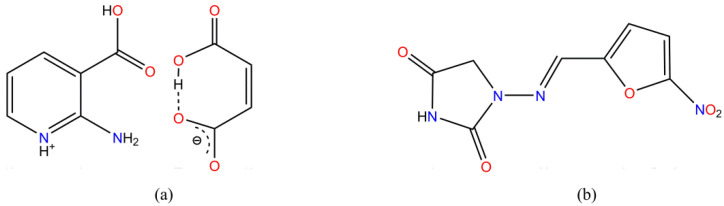
Structures of salts of protonated 2-amino-nicotinic acid and maleic acid monoanion (**a**) and *N*-(5-Nitro-2-furfurylidene)-1-aminohydantoin (**b**).

1. The features of H-bonded networks in hydrates and isomorphic peroxosolvates of multicomponent organic crystals were identified using the Cambridge Structural Database version 5.42 (September 2021) [33] and version 2016-1 of the Inorganic Crystal Structure Database [34].
2. The H-bond enthalpy in crystalline hydrates ([2-amino-nicotinic acid+maleic acid+H_2_O] (1:1:1), [*N*-(5-Nitro-2-furfurylidene)-1-aminohydantoin+H_2_O] (1:1)) and peroxosolvates ([2-amino-nicotinic acid+maleic acid+H_2_O_2_] (1:1:1), [*N*-(5-Nitro-2-furfurylidene)-1-aminohydantoin+H_2_O_2_] (1:1)) (Scheme 1) was determined using periodic DFT calculations [30] followed by Rosenberg’s equation [23,24]. The structures of the crystalline hydrates were studied by X-ray diffraction [35,36]. The isomorphic or isostructural peroxosolvates were purposefully prepared for this study.
3. The spectroscopic features of the considered crystals were studied by low-frequency Raman spectroscopy followed by periodic DFT computations.

## 2. Results and Discussion

### 2.1. Features of H-Bonded Networks in Hydrates and Isomorphic Peroxosolvates

Our statistics are based on 103 peroxosolvates, 91 of which are organic crystals’ structures [33,34]. The detailed analysis of these crystals’ structures is given in Tables S1 and S2 of Ref. [28]. After excluding structures with structurally disordered H_2_O_2_ molecules and structures with non-alkaline metals, the number of peroxosolvates decreased to 56. Furthermore, those structures were excluded in which H_2_O_2_ molecules interact directly with the Li^+^, Na^+^, K^+^, NH_4_^+^ ions or with other H_2_O_2_ molecules through H-bonds. The crystals containing the other solvent molecules were also excluded from consideration. As a result, the final number of analyzed peroxosolvates was 46. We concluded the following: (1) H_2_O_2_ does not form H-bonds as a proton acceptor if the organic coformers do not have active hydrogen atoms. Such crystals make up a significant proportion of the considered structures (20). (2) H_2_O_2_ forms three or four H-bonds as a proton acceptor in five structures. The oxygen atoms of H_2_O_2_ are less likely to participate in bifurcate H-bonds than the C=O and P=O groups [20,37]. (3) In the remaining structures (21), the H_2_O_2_ molecule forms one or two bonds as a proton acceptor. If the coformer is not a zwitterion, then H_2_O_2_ can form the quasi-linear O∙∙∙H-N bonds (Figure S2). (4) Currently, there is only one example of the bifurcate H-bond formed by the OH group of H_2_O_2_ [38]. Refcodes of the analyzed peroxosolvates are given in Table S2. Analysis of secondary interactions, in particular, O∙∙∙H-C bonds [39,40], is beyond the scope of this work.

The comparison of the metric and energy characteristics of H-bonds formed by the H_2_O_2_ and H_2_O molecules in the solid state involves the use of isomorphic peroxosolvates and crystalline hydrates. Notably, the number of such structures is not large. By means of a special choice of coformers (cyclic N-oxides), isomorphic crystal hydrates and peroxosolvates containing only two H-bonds formed by H_2_O and H_2_O_2_ as a proton donor were studied in ref. [10]. It was stated that H_2_O_2_ forms shorter H-bonds as compared to H_2_O. A similar trend was previously revealed in refs. [15,32] in isomorphic crystalline hydrates and peroxosolvates of several amino acids. These studies show that the hydrogen peroxide molecule usually forms shorter (strong) donor H-bonds than the water molecule. Data on the proton acceptor properties of H_2_O_2_ and H_2_O in the solid state are scarce. The values of the O∙∙∙H-N^+^ bond energy are comparable in isomorphic crystallohydrate and peroxosolvate of serine [15]. The O∙∙∙H-N bond energy in the crystalline hydrate of the cyclic dipeptide is much higher than that in the isomorphic peroxosolvate [32]. This issue required an additional study, which was performed in the next section.

### 2.2. The H-Bond Enthalpy in the Selected Crystalline Hydrates and Peroxosolvates

**Figure 1:**
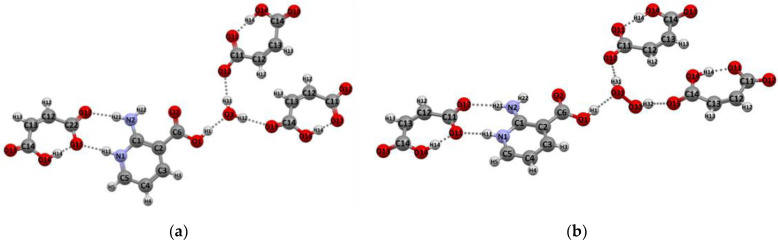
The fragments of the [2AmNic+Mle+H_2_O] [36] (**a**) and [2AmNic+Mle+H_2_O_2_] (**b**) structures. H-bonds are denoted by dotted lines.

**Figure 2:**
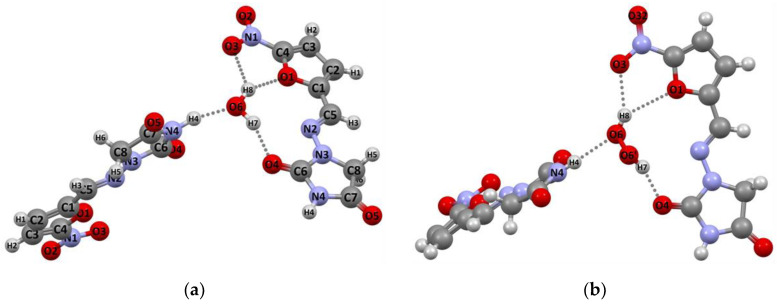
The fragments of the [NFA+H_2_O] [35] (**a**) and [NFA+H_2_O_2_] (**b**) structures. H-bonds are denoted by dotted lines.

Crystalline [2-amino-nicotinic acid+maleic acid+H_2_O] (1:1:1), denoted below as [2AmNic+Mle+H_2_O], is a relevant object for the following reasons. Firstly, H_2_O forms three quasi-linear H-bonds in this crystal—two as a proton donor and one as a proton acceptor [36]. Secondly, the structure, the spectrum, and the *E*_HB_/*H* values of H-bond in this crystal were characterized by X-ray analysis, terahertz Raman spectroscopy, and periodic DFT calculations [36]. In this work, we accomplished the synthesis of peroxosolvate [2AmNic+Mle+H_2_O_2_] (1:1:1) (Section 3.3). The network of H-bonds formed by H_2_O_2_ in this crystal is equivalent to the network of H-bonds formed by H_2_O in crystalline (cf. [2AmNic+Mle+H_2_O_2_] and [2AmNic+Mle+H_2_O], Figure 1). These crystals are isostructural. Crystalline [*N*-(5-Nitro-2-furfurylidene)-1-aminohydantoin+H_2_O] (1:1) [35], denoted below as [NFA+H_2_O] (Figure 2), was chosen as the second model of crystallohydrate. In this crystal, the H_2_O molecule forms one quasi-linear H-bond as a proton acceptor and three H-bonds as a proton donor, with one of the OH groups forming a bifurcate bond. Unlike [2AmNic+Mle+H_2_O] crystal, in the [NFA+H_2_O] crystal a water molecule forms H-bonds as a proton donor with the same neutral molecule, whereas H-bond as a proton acceptor is formed with the H-N group of a neighboring molecule. Moreover, we obtained peroxosolvate [NFA+H_2_O_2_] (1:1), isomorphic to this crystalline hydrate (Figure 2). The network of H-bonds in the synthesized [NFA+H_2_O_2_] is presented in Figure 2b.

The theoretical values of the enthalpy of intermolecular H-bonds in the considered crystals were evaluated using the equation (1), where the O∙∙∙H distances were calculated at the PBE-D3/6-31G** level (Table 1). In accordance with the literature data [30,31] the relaxed values of the O∙∙∙H distance in molecular crystals systematically exceed the X-ray values by ~0.15 Å. The deviations from this parameter are due to serious differences between the theoretical distances A∙∙∙B and the experimental ones.

To estimate the error in determining the ∆*H*_HB_ parameter caused by a significant deviation of the theoretical *R*(H∙∙∙O) values from the experimental ones, the *R*(H∙∙∙O) values were computed at the B3LYP/6-31G** level (Tables S2 and S3). The Δ*H_HB_* values calculated at the B3LYP/6-31G** level agree nicely with those obtained using the PBE-D3/6-31G** level (cf. Table 1 with Tables S2 and S3). In accordance with the literature [41,42], the PBE-D3 calculations slightly overestimate the H-bonded energy compared to the B3LYP calculations.

The enthalpies of H-bonds formed by H_2_O_2_ as a proton donor turned out to be higher or comparable with the values of the corresponding H-bonds formed by H_2_O [15,32,43]. In the case of H-bonds formed by H_2_O_2_ and H_2_O as a proton acceptor, the picture is reversed. In accordance with the literature data [32], the enthalpy of H-bonds formed by H_2_O as a proton acceptor is systematically higher than the analogous values of H-bonds formed by H_2_O_2_.

As it follows from the data (Table 1), the value of the enthalpy of the bifurcate H-bond formed by the OH group is significantly higher than the enthalpy of the ordinary H-bond formed by another OH group of H_2_O or H_2_O_2_.

### 2.3. Low-Frequency Raman Spectra of the Considered Crystals

**Figure 3:**
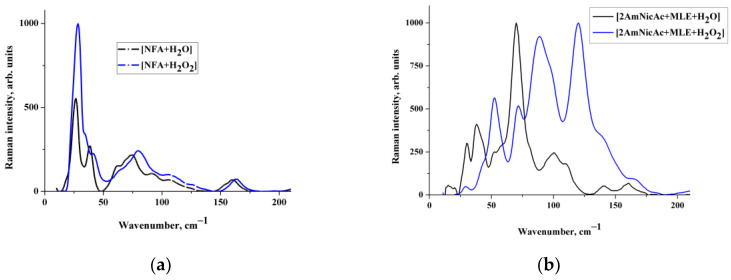
The experimental low-frequency Raman spectra of the [NFA+H_2_O] and [NFA+H_2_O_2_] complexes (**a**); the [2AmNic+Mle+H_2_O] and [2AmNic+Mle+H_2_O_2_] complexes (**b**).

Low-frequency Raman spectroscopy is widely used in the investigation of organic materials [44]. A special attention is paid to intermolecular interactions [45,46,47], in particular, H-bonds [48]. We showed that B3LYP and PBE-D3 with fixed cell parameters provide a reasonable description of the low-frequency Raman spectra of the multicomponent molecular crystals [26,49]. In this work, the Raman spectra of the considered crystals were investigated in the 10–2000 cm^−1^ frequency region for two purposes (Figure 3). Firstly, to identify possible differences in the low-frequency Raman spectra of crystallohydrates and isomorphous peroxolvates. Secondly, to find out how well the periodic DFT computations with the 6-31G** basis set reproduce the Raman spectra of the considered crystals in the mid-frequency range.

Crystalline [NFA+H_2_O] is isomorphic to crystalline [NFA+H_2_O_2_] (Table 2). Their experimental low-frequency spectra are very similar (Figure 3a). The situation is different for crystalline [2AmNic+Mle+H_2_O] and crystalline [2AmNic+Mle+H_2_O_2_] (Figure 3b). This result can be explained by the fact that the latter crystals have a similar network of H-bonds, but their crystal structure is different (Table 2 and data from refs [35,36]).

**Figure 4:**
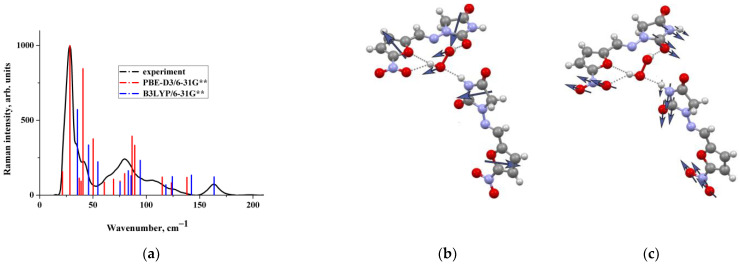
The experimental (black line) and calculated (PBE-D3—red sticks and B3LYP—blue sticks) low-frequency Raman spectra of the [NFA+H_2_O_2_] crystal (**a**). The height of the bars is proportional to the relative Raman intensity of corresponding transition. The schematic representation of the atom displacements of the two Raman intense vibrations (PBE-D3/6-31G**) at 30 cm^−1^ (**b**) and 80 cm^−^^1^ (**c**).

**Figure 5:**
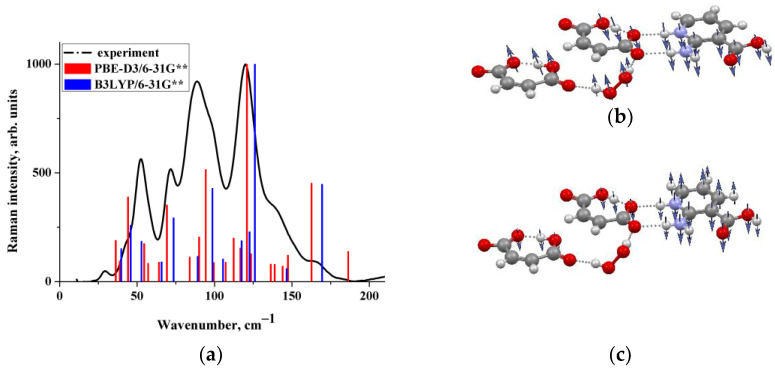
The experimental (black line) and calculated (PBE-D3—red sticks and B3LYP—blue sticks) low-frequency Raman spectra of the [2AmNic+Mle+H_2_O_2_] crystal (**a**). The theoretical values of wavenumbers are scaled by 0.9. The height of the bars is proportional to the relative Raman intensity of the corresponding transition. The schematic representation of the atom displacements of the two Raman intense vibrations (PBE-D3/6-31G**) at 44 cm^−1^ (**b**) and 94 cm^−^^1^ (**c**).

The PBE-D3/6-31G** and B3LYP/6-31G**calculations describe the low-frequency Raman spectra of crystalline [NFA+H_2_O_2_] (Figure 4). The computed wavenumbers of the most bands are bathochromically shifted, which is a common occurrence for harmonic frequencies of molecular crystals calculated using all-electronic Gaussian-type orbital bases [50]. The most intense band (~30 cm^−1^) is characterized by the essential displacements of the oxygen atoms of H_2_O_2_ and the librations (rotations) of the five-member rings of NFA. The atomic displacements of the H_2_O_2_ molecule are negligible in the band around 80 cm^−1^ (c.f. Figure 4b,c). The low-frequency Raman spectra of crystalline [NFA+H_2_O] are compared with the theoretical ones in Figure S3. Such as in the case of the isomorphic peroxosolvate, the intense band of the water molecule vibrations did not occur in the low region (Figure S4a,b). Therefore, low-frequency Raman spectroscopy seems to be hardly applicable to distinguish between the crystalline hydrates of NFA. Similar results were obtained for [2AmNic+Mle+H_2_O_2_] and [2AmNic+Mle+H_2_O]. In these crystals, no intense Raman band was observed, which could be assigned to vibrations of H_2_O_2_ (Figure 5b,c) or H_2_O (Figure S6a,b).

In the three crystals studied, the theoretical spectrum is in good agreement with the experimental one (Figure 4a, Figures S3 and S5). To harmonize the agreement between the experimental and theoretical Raman spectra of crystalline [2AmNic+Mle+H_2_O_2_] in the low-frequency region, a scaling factor of 0.9 was used (Figure 5a). Possible reasons for using a scaling factor for a single crystal are as follows. Scaling factors were developed for isolated molecules simpler than molecular crystals [51]. The low-frequency Raman-active vibrations in molecular crystals are mainly associated with the librational motions (Figure 1 in [52]). An additional reason for the difference between the calculated and experimental frequencies in the region below 200 cm^−1^ may be crystal packing effects. In the case of the studied isomorphic crystals, this error is the same for [NFA+H_2_O] and [NFA+H_2_O_2_]. Crystals of 2-amino-nicotinic acid have different symmetry groups (Table 2), and the error in the calculation of the wavenumbers of low-frequency vibrations differs for [2AmNic+Mle+H_2_O] and [2AmNic+Mle+H_2_O_2_].

The O–O stretching vibration of H_2_O_2_ is located at 870 cm^−1^ in crystalline peroxosolvates [53,54]. To check the stability of the considered peroxosolvates after laser excitation during Raman measurements, the obtained spectra were investigated in the frequency range of 850–950 cm^−1^ and compared with the results of periodic DFT calculations (Figures S7 and S8). The experimental spectra of crystalline [NFA+H_2_O_2_] and crystalline [2AmNic+Mle+H_2_O_2_] do have Raman active bands around 880–890 cm^−1^. The calculated vibration at 910 cm^−1^ in both theoretical spectra corresponds to the O–O stretching vibration of H_2_O_2_.

## 3. Materials and Methods

### 3.1. Compounds and Solvents

Anhydrous NFA (CAS no. 67-20-9) with purity of 98% was purchased from Sigma-Aldrich (St. Louis, MO, USA) and used without additional purification. The 2-aminonicotinic acid (5345-47-1, 98%) was purchased from Sigma-Aldrich, and the maleic acid (110-16-7, 98%) was bought from Merck KGaA (Darmstadt, Germany).

The salt hydrate [2AmNic+Mle+H_2_O] (1:1:1) was obtained according to the method described in the work by Surov et al. [36]. A stoichiometric mixture of 2AmNic and Mle was suspended in water to form a slurry and stirred on a magnetic stirrer overnight.

96% hydrogen peroxide. Danger of explosion! 100 mL of laboratory reagent grade 60% H_2_O_2_ (Fisher Scientific, Loughborough, UK) was concentrated to approximately 25 mL with an exhaustively cleaned rotary evaporator without the use of any vacuum grease (temperature exactly 50 °C and pressure 8 mbar). The concentration of the residual solution was examined by refractometry (*n*_D_ = 1.403 at 20°) [55].

### 3.2. Cocrystal Preparation

[2AmNic+Mle] and [NFA] were being dried in a treating oven for 5 h (80 °C, 100 mbar). 50 mg of unsolvated [2AmNic+Mle] or [NFA] were put into 2 mL vial and 1 mL of 96% hydrogen peroxide was added. The vials were tightly capped. The starting materials were dissolved by an intensive shaking of the vials at 50 °C. The obtained transparent solutions were stored in the freezer for one week at −23 °C. In both cases, high quality crystals (with dimensions up to 0.5 mm) were obtained. Afterwards, the surplus mother liquids were immediately removed from the cold vials using Pasteur pipets.

### 3.3. Single Crystal X-ray Diffraction

The diffraction intensities for [2AmNic+Mle+H_2_O_2_] and [NFA+H_2_O_2_] were collected on a Bruker D8 Venture machine (Bruker AXS, Karlsruhe, Germany) using graphite monochromatized Mo*K*_α_ radiation (λ = 0.71073 Å) at 100 and 150 K, respectively. The absorption corrections based on measurements of equivalent reflections were applied [56]. The structures were solved by direct methods and refined by full matrix least-squares on *F*^2^ with anisotropic thermal parameters for all non-hydrogen atoms [57]. All hydrogen atoms were found from difference Fourier synthesis and refined isotropically. The experimental details are listed in Table 2.

The crystallographic data for [2AmNic+Mle+H_2_O_2_] and [NFA+H_2_O_2_] were deposited with the Cambridge Crystallographic Data Centre as supplementary publications under the CCDC numbers 2115827 and 2115828, respectively.

### 3.4. Raman Spectroscopy

The Raman measurements in the spectral range of 10−2000 cm^−1^ were conducted using a Raman microscope (inVia, Renishaw plc, Spectroscopy Product Division, Old Town Wotton-Under-Edge, Gloucestershire, UK) with the 50× objective lens (Leica DM 2500 M, NA = 0.75, Leica Mikrosysteme Vertrieb gmbH Mikroskopie und HistologieErnst-Leitz-Strasse 17-37, Wetzlar, Germany). The measurements in the spectral range of 10−200 cm^−1^ were made with a NExT monochromator, whereas in the spectral range 200−2000 cm^−1^ they were made with an edge-filter. The excitation wavelength was 633 nm, being provided by a He-Ne laser (RL633, Renishaw) with the maximum power of 17 mW. The acquisition time and number of accumulations were adjusted to maximize the signal-to-noise ratio with the minimal sample degradation. All the spectra for the powder samples were measured at several points and then averaged to reduce the anisotropy effect on the Raman spectra and to increase the single-to-noise ratio. The background from the Raman spectra was subtracted by the cubic spline interpolation method. All the spectra were divided by the number of accumulations and acquisition time.

### 3.5. Periodic (Solid-State) DFT Computations

The Kohn–Sham methods with periodic boundary conditions (periodic DFT) provide a grounded trade-off between the accuracy and the rate of calculations of experimentally observed properties of multi-component organic crystals [58,59,60]. The computations with all-electron Gaussian-type localized orbital basis 6-31G** were conducted using the CRYSTAL17 package [61]. B3LYP [62,63] and PBE [64] were employed. The London dispersion interactions were taken into account by introducing the D3 correction with Becke-Jones damping (PBE-D3) developed by Grimme et al. [65]. The space groups and the unit cell parameters of the crystals obtained from the X-ray diffraction experiment were fixed and the structural relaxations were limited to the positional parameters of the atoms (AtomOnly). Further details of the periodic DFT calculations are given in the Supporting Information.

## 4. Conclusions

According to the structural databases, there are 103 peroxosolvates, 91 of which are organic crystals. After excluding crystals with structurally disordered H_2_O_2_ molecules, crystals in which H_2_O_2_ molecules directly interact with the Li^+^, Na^+^, K^+^, NH_4_^+^ ions or with other H_2_O_2_ molecules, the number of analyzed peroxosolvates decreased to 38. In these crystals, H_2_O_2_ interacts with the surrounding organic molecules through H-bonds. We concluded the following: (i) H_2_O_2_ does not form classical H-bonds as a proton acceptor if the organic coformers do not have active hydrogen atoms. Such crystals make up a significant proportion of the considered crystals (43%). (ii) H_2_O_2_ forms three or four H-bonds as a proton acceptor in five crystals. The oxygen atoms of H_2_O_2_ are less likely to participate in bifurcate H-bonds than the C=O and P=O groups. (iii) In the remaining 46% of crystals, the H_2_O_2_ molecule forms one or two bonds as a proton acceptor. (iv) Currently, there are only a few examples of a bifurcate H-bond formed by the OH group of H_2_O_2_. These findings allow suggesting that suitable H-bond acceptors in the coformer molecule are crucial for peroxosolvate formation, while the presence of strong H-bond donors is not mandatory.

In accord with the literature [15,16], the enthalpies of H-bonds formed by H_2_O_2_ as a proton donor turned out to be higher than the values of the corresponding H-bonds formed by H_2_O in the considered crystals. The enthalpy of the bifurcate H-bond formed by the OH group is significantly higher than the enthalpy of the ordinary H-bond formed by another OH group of H_2_O or H_2_O_2_. The proton acceptor properties of H_2_O are stronger than those of H_2_O_2_. The neutral O∙∙∙H-O/O∙∙∙H-N bonds formed by the lone electron pair of the oxygen atom of water turned out to be the strongest H-bonds in the considered crystals.

The bands in the low-frequency Raman spectra of the considered crystals, characterized exclusively by the vibrations of the atoms of H_2_O_2_ or water molecules, were not traced. Some Raman intense bands below 50 cm^−1^ are characterized by the essential displacements of the oxygen atoms of H_2_O_2_ or H_2_O. The displacement of the H_2_O_2_ molecule is negligible in the lattice vibrations [54] located in the 50–150 frequency region.

## Figures and Tables

**Table 1:** Distances between the atoms involved in the formation of intermolecular H-bonds in [2AmNic+Mle+H_2_O], [2AmNic+Mle+H_2_O_2_], [NFA+H_2_O] and [NFA+H_2_O_2_]; *R*(O∙∙∙N), *R*(O∙∙∙O), and *R*(H∙∙∙O) obtained using periodic DFT computations (PBE-D3/6-31G**) and the ∆*H*_HB_ values evaluated using Equation (1).

Fragment ^1^	*R*(O∙∙∙N)/*R*(O∙∙∙O) ^2^, Å	*R*(H∙∙∙O), Å	∆*H*_HB_, kJ/mol
[2AmNic+Mle+H_2_O] (1:1:1)			
O12…H21-N2	2.804 (2.816)	1.777 (1.928)	26.0
O11…H11-N1	2.816 (2.816)	1.770 (1.905)	26.4
O12…H31-O3	2.701 (2.717)	1.707 (1.860)	29.4
O13…H32-O3	2.765 (2.771)	1.783 (1.900)	25.8
O3…H1-O1	2.536 (2.567)	1.484 (1.641)	45.1
[2AmNic+Mle+H_2_O_2_] (1:1:1)			
O12…H21-N2	2.846 (2.831)	1.810 (1.955)	24.6
O11…H11-N1	2.849 (2.735)	1.805 (1.803)	24.8
O12…H31-O31	2.658 (2.636)	1.648 (1.759)	32.8
O13…H32-O32	2.774 (2.698)	1.841 (1.767)	23.3
O31…H1-O1	2.726 (2.646)	1.738 (1.769)	27.9
[NFA+H_2_O]			
N4-H4…O6	2.689 (2.763)	1.653 (1.782)	32.5
O6-H7…O4	2.834 (2.961)	1.938 (2.015)	20.0
O6-H8…O1	3.080 (3.148)	2.338 (2.245)	11.3
O6-H8…O3	2.966 (3.172)	2.100 (2.392)	15.6
[NFA+H_2_O_2_]			
N4-H4…O6	2.807 (2.905)	1.822 (2.098)	24.1
O6’-H7…O4	2.735 (2.737)	1.786 (1.894)	25.6
O6-H8…O1	3.175 (3.128)	2.496 (2.477)	9.2
O6-H8…O3	2.871 (2.907)	1.935 (2.144)	20.1

^1^ the atomic numbering is given in Figure 1 and Figure 2; ^2^ experimental values are given in parentheses.

**Table 2:** Crystal data and refinement details of the [2AmNic+Mle+H_2_O_2_] and [NFA+H_2_O_2_] structures.

	[2AmNic+Mle+H_2_O_2_]	[NFA+H_2_O_2_]
Empirical formula	C_10_H_12_N_2_O_8_	C_8_H_8_N_4_O_7_
*F*w	288.22	272.18
color, habit	colorless, prism	light-yellow, prism
crystal size (mm)	0.25 × 0.20 × 0.15	0.25 × 0.15 × 0.10
crystal system	monoclinic	orthorhombic
space group	*P*2_1_/n	*P*bca
*a* (Å)	9.5451(4)	13.0154(7)
*b* (Å)	11.7871(4)	9.4659(7)
*c* (Å)	11.0780(4)	17.9512(10)
*β* (deg)	105.089(1)	90
*V* (Å^3^)	1203.40(8)	2211.6(2)
*Z*	4	8
*D*_c_ (g·cm^−3^)	1.591	1.635
*μ* (mm^−1^)	0.140	0.146
*F*(000)	600	1120
*θ* range (deg)	2.51 to 29.00	2.27 to 28.00
refl collcd	11941	19769
indep reflns/*R*_int_	3189/0.0219	2671/0.0650
reflns *I* > 2σ(*I*)	2742	1995
No of param	229	205
GooF on *F*^2^	1.047	1.025
*R*_1_ (*I* > 2σ(*I*))	0.0345	0.0371
*wR*_2_(all data)	0.0954	0.0882
largest diff peak/hole (e·Å^−3^)	0.368/−0.191	0.250/−0.244

## Data Availability

The Raman spectra and I/O files are available from the respective author upon reasonable request.

## Supplementary Materials

The following are available online, Figure S1: The fragment of crystalline L-Serine hydrogen peroxide solvate (CCDC 726697) [17], Figure S2: The fragment of crystalline 5,5′-dinitro-2H,2′H-3,3′-bi-1,2,4-triazole hydrogen peroxide solvate (CCDC 1874657) [66], Table S1: Refcodes of the analyzed peroxosolates, Figure S3: Low-frequency Raman spectrum of crystalline [NFA+H_2_O]. Experiment vs. PBE-D3/6-31G** computations, Figure S4: Schematic representation of atom displacements of the two Raman intense vibrations (PBE-D3/6-31G**) of crystalline [NFA+H_2_O] around 16 cm^−1^ (**a**); and 64 cm^−^^1^ (**b**). Figure S5: Low-frequency Raman spectrum of crystalline [2AmNic+Mle+H_2_O]. Experiment vs. B3LYP/6-31G** computations, Figure S6: Schematic representation of atom displacements of the two Raman intense vibrations (B3LYP/6-31G**) of crystalline [2AmNic+Mle+H_2_O] around 50 cm^−^^1^ (**a**); and 72 cm^−^^1^ (**b**), Figure S7: Raman spectrum of crystalline [NFA+H_2_O_2_] in the 850–950 frequency region. Experiment vs. PBE-D3 computations, Figure S8: Raman spectrum of crystalline [2AmNic+Mle+H_2_O_2_] in the 850–950 frequency region. Experiment vs. PBE-D3 computations, Section S1: Details of the periodic DFT calculations, Table S2: Distances between the atoms involved in the formation of intermolecular H-bonds in [2AmNic+Mle+H_2_O] (1:1:1) and [2AmNic+Mle+H_2_O_2_] (1:1:1), *R*(O∙∙∙N), *R*(O∙∙∙O), and *R*(H∙∙∙O) obtained using periodic DFT computations (B3LYP/6-31G**) and the ∆*H*_HB_ values evaluated using Equation (1), Table S3: Distances between the atoms involved in the formation of intermolecular H-bonds in [NFA+H_2_O] and [NFA+H_2_O_2_], *R*(O∙∙∙N), *R*(O∙∙∙O), and *R*(H∙∙∙O) obtained using periodic DFT computations (B3LYP/6-31G**) and the ∆*H*_HB_ values, evaluated using Equation (1).

## Sample Availability

No possibility.
