# Supplementary material for: Comparison of Proton Acceptor and Proton Donor Properties of H2O and H2O2 in Organic Crystals of Drug-like Compounds: Peroxosolvates vs. Crystallohydrates
Source: Molecules. 2022 Jan 22;27(3):717. doi: 10.3390/molecules27030717 (PMC8838768; doi:10.3390/molecules27030717)
Supplement: Supplementary file 1 [file molecules-27-00717-s001.zip › molecules-1514202-supplementary.pdf]

Supporting Information for Publication

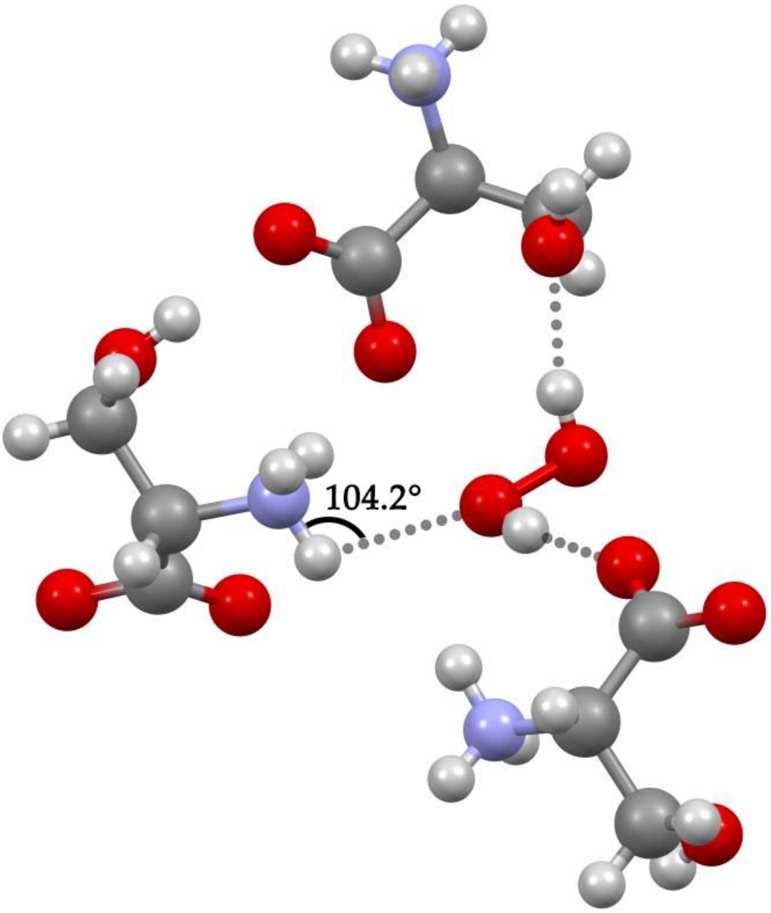

Figure S1. The fragment of crystalline L-Serine hydrogen peroxide solvate (CCDC 726697) [s1]. H-bonds are denoted by dotted lines.

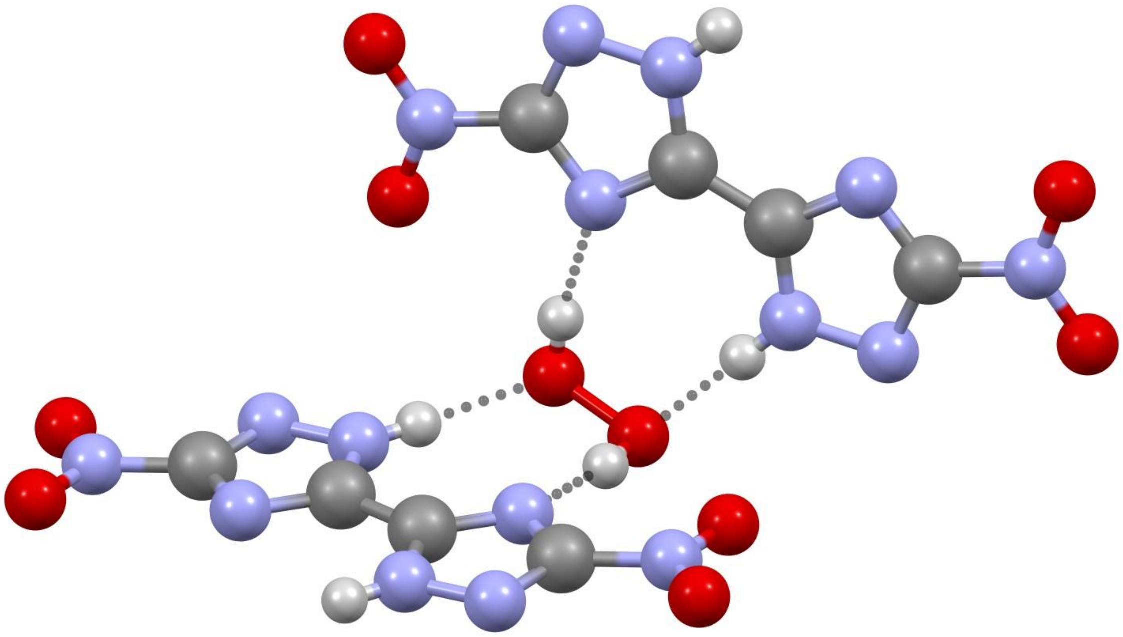

Figure S2. The fragment of crystalline 5,5’-dinitro-2H,2’H-3,3’-bi-1,2,4-triazole hydrogen peroxide solvate (CCDC 1874657) [s2]. H-bonds are denoted by dotted lines.

Table S1. Refcodes of peroxosolvates analyzed in Section 2.1, grouped by the number of conventional H-bonds formed by the H_2O_2 molecule as a proton acceptor (AH-bonds).

Number of AH-bonds	0	1	2	3	4
Refcodes	CAZHUH<br>TOYTEJ<br>POMQIU<br>POMQUG<br>BAFGOH<br>VANVOX<br>XETSUK<br>DOJMIZ<br>JELQOJ<br>JESXEN<br>KUMRER<br>MUXHIX<br>RIKJAW<br>UKEFEV<br>UDUROD<br>BONGES<br>BONGIW<br>TANCAO<br>ANINAO<br>HOQSEO	BOHLOC<br>TANDAP<br>VILFUU<br>ANINES<br>HOQSIS<br>ANIMUH	VAYGUY<br>VAYGUY01<br>JOZZED<br>KULMOU<br>TANCIW<br>TANCOC<br>TANCES	TANDET<br>KULMUA	UREXPO11<br>YAFFUJ<br>OHIJEX

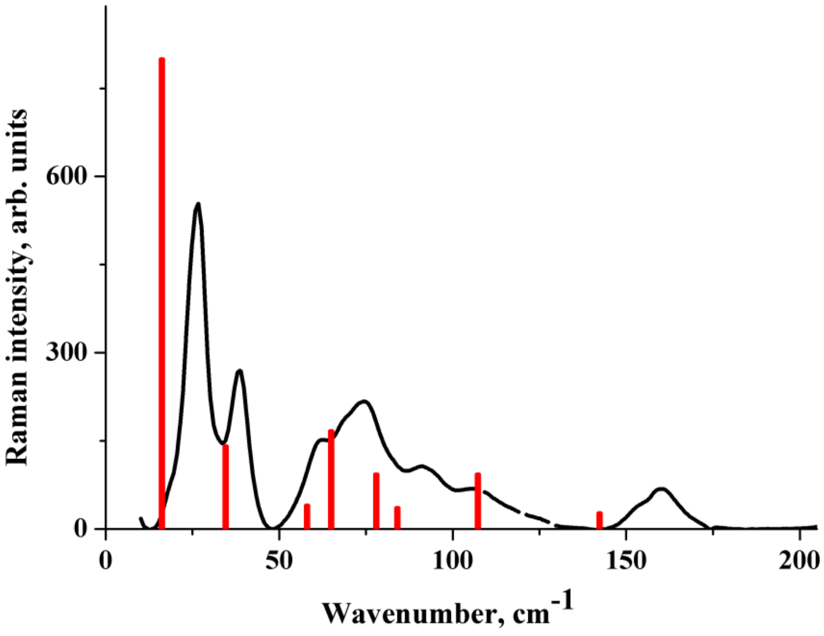

Figure S3. Low-frequency Raman spectrum of crystalline [NFA+H_2O]. Experiment (black line) vs. PBE-D3/6-31G** computations (red sticks). The height of the bars is proportional to the relative Raman intensity of the corresponding transition.

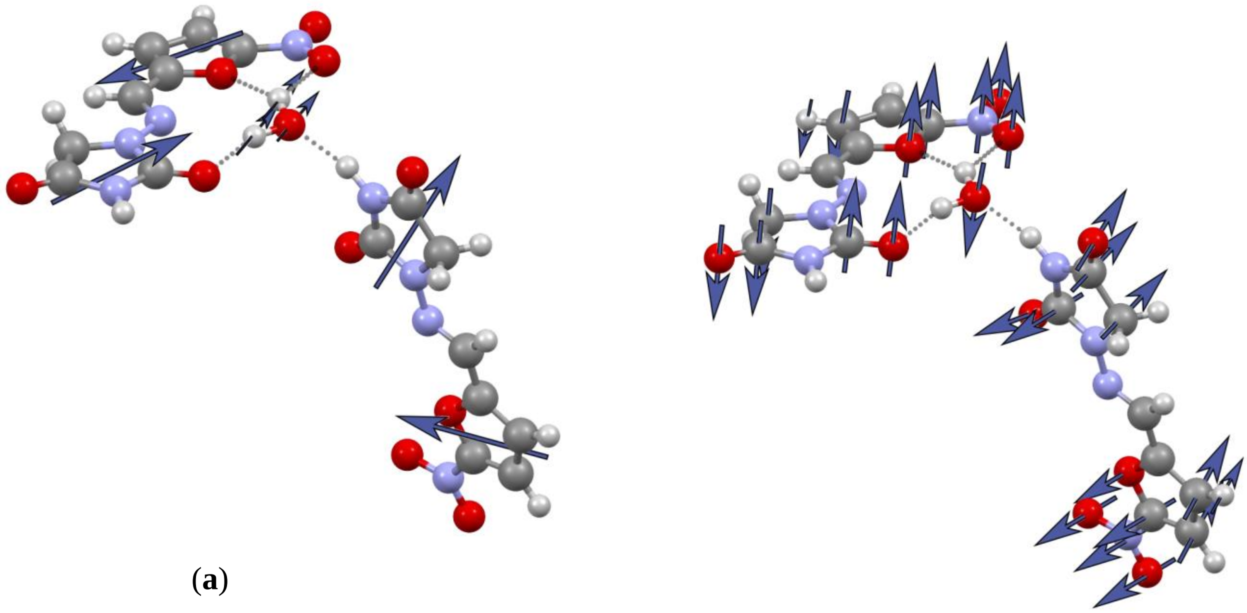

(**a**)

(**b**)

Figure S4. The schematic representation of atom displacements of the two Raman intense vibrations (PBE-D3/6-31G**) of crystalline [NFA+H_2O] around 16 cm^{-1} (**a**); and 64 cm^{-1} (**b**).

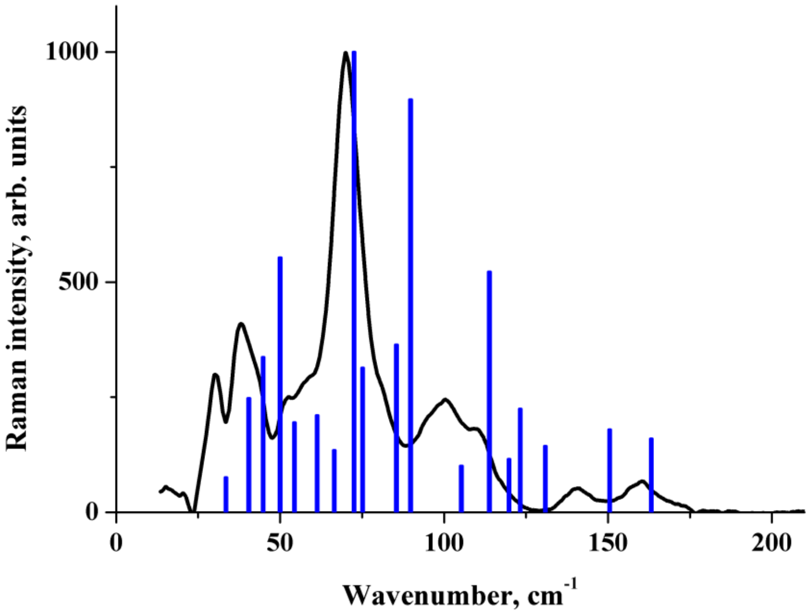

Figure S5. Low-frequency Raman spectrum of crystalline [2AmNic+Mle+H_2O]. Experiment (black line) vs. B3LYP/6-31G** computations (blue sticks). The height of the bars is proportional to the relative Raman intensity of the corresponding transition. The scaling factor is not used in this Figure.

(**a**)

(**b**)

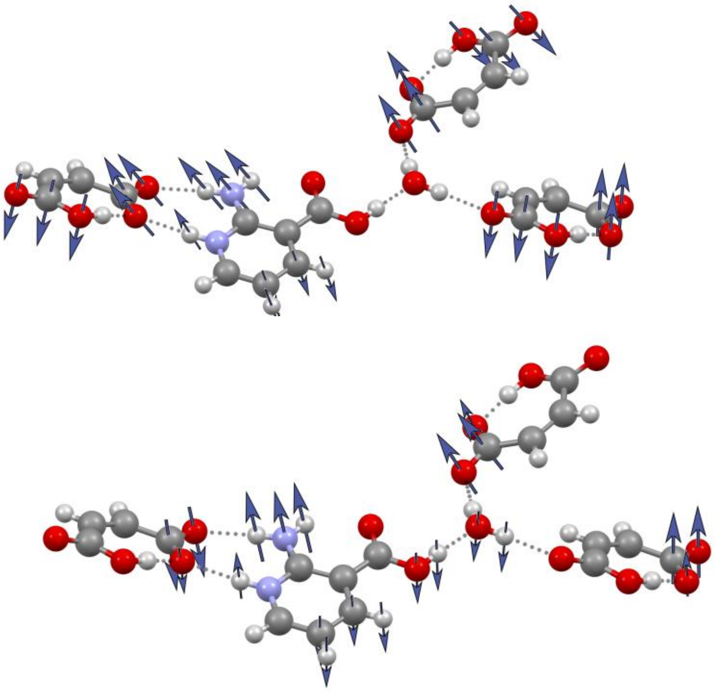

Figure S6. The schematic representation of atom displacements of the two Raman intense vibrations (B3LYP/6-31G**) of crystalline [2AmNic+Mle+H_2O] around 50 cm^{-1} (**a**); and 72 cm^{-1} (**b**).

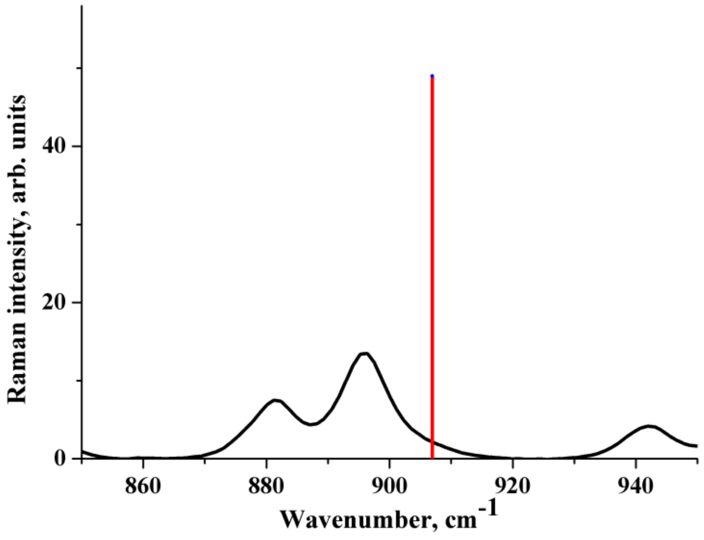

Figure S7. Raman spectrum of crystalline [NFA+H_2O_2] in the 850 – 950 frequency region. Experiment (black line) vs. PBE-D3 computations (red sticks). The scaling factor is not used in this Figure. The height of the sticks is proportional to the relative Raman intensity of the corresponding transition. The experimental and theoretical spectra are normalized to the maximal intensity, which equals 1000 arb. units.

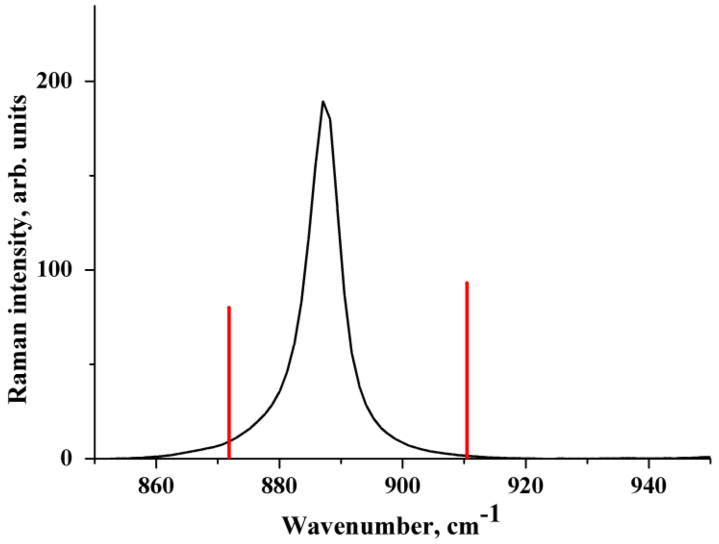

Figure S8. Raman spectrum of crystalline [2AmNic+Mle+H_2O_2] in the 850 – 950 frequency region. Experiment (black line) vs. PBE-D3 computations (red sticks). The scaling factor is not used in this Figure. The height of the sticks is proportional to the relative Raman intensity of the corresponding transition. The experimental and theoretical spectra are normalized to the maximal intensity, which equals 1000 arb. units.

S1. Details of the periodic DFT calculations.

Tolerances on energies which control the self-consistent field convergence for geometry optimizations and frequency computations were set to 1×10^{-8} and 1×10^{-11} Hartree, respectively. The shrinking factor of the reciprocal space net was set to 3. Frequencies of normal modes were calculated within the harmonic approximation. IR and Raman intensities were obtained via a coupled perturbed Hartree-Fock (CPHF) [s3, s4]. The number of points in the numerical first-derivative calculation of the analytic nuclear gradients equals 2. Raman intensities were calculated using the “RAMANEXP” keyword. Temperature was 298 K, the frequency of the incoming laser was 633 nM.

Table S2. Distances between the atoms involved in the formation of intermolecular H-bonds in [2AmNic+Mle+H_2O] (1:1:1) and [2AmNic+Mle+H_2O_2] (1:1:1), *R*(O…N), *R*(O…O), and *R*(H…O) obtained using periodic DFT computations (B3LYP/6-31G**) and the ΔH_{HB} values evaluated using Eq. (1).

Fragment [a]	*R*(O…N)/*R*(O…O)[b], Å	*R*(H…O), Å	ΔH_{HB} , kJ/mol
[2AmNic+Mle+H_2O] (1:1:1)			
O12…H21-N2	2.811 (2.816)	1.792	25.4
O11…H11-N1	2.818 (2.816)	1.785	25.7
O12…H31-O3	2.706 (2.717)	1.725	28.5
O13…H32-O3	2.706 (2.771)	1.797	25.2
O3…H1-O1	2.550 (2.567)	1.525	41.5
[2AmNic+Mle+H_2O_2] (1:1:1)			
O12…H21-N2	2.860 (2.831)	1.836	23.6
O11…H11-N1	2.873 (2.735)	1.841	23.4
O12…H31-O3	2.668 (2.636)	1.674	31.2
O13…H32-O3’	2.769 (2.698)	1.849	23.1
O3…H1-O1	2.757 (2.646)	1.786	25.6

[a] the atomic numbering is given in Figs. 1; [b] experimental values are given in parentheses.

Table S3. Distances between the atoms involved in the formation of intermolecular H-bonds in [NFA+H_2O] and [NFA+H_2O_2], *R*(O…N), *R*(O…O), and *R*(H…O) obtained using periodic DFT computations (B3LYP/6-31G**) and the ΔH_{HB} values, evaluated using Eq. (1).

Fragment [a]	*R*(O…N)/*R*(O…O)[b], Å	*R*(H…O), Å	ΔH_{HB}, kJ/mol
[NFA+H_2O]			
N4-H4…O6	2.734 (2.763)	1.708	29.4
O6-H7…O4	2.880 (2.961)	1.983	18.7
O6-H8…O1	3.168 (3.148)	2.392	10.5
O6-H8…O3	3.038 (3.172)	2.185	13.9
[NFA+H_2O_2]			
N4-H4…O6	2.843 (2.905)	1.872	22.2
O6'-H7…O4	2.731 (2.737)	1.788	25.6
O6-H8…O1	3.227 (3.128)	2.525	8.9
O6-H8…O3	2.888 (2.907)	1.977	18.8

[a] the atomic numbering is given in Figs. 2; [b] experimental values are given in parentheses.

References.

s1) A. V. Churakov, P. V. Prikhodchenko, J. A. K. Howard, O. Lev, Chem. Commun. 4224 (2009).

s2) Ren A. Wiscons, Michael K. Bellas, Jonathan C. Bennion, Adam J. Matzger, Cryst. Growth Des. 18, 7701 (2018).

s3) L. Maschio, B. Kirtman, R. Orlando, M. Rèrat, J. Chem. Phys. 137, 204113 (2012).

s4) L. Maschio, B. Kirtman, M. Rerat, R. Orlando, R. Dovesi, J. Chem. Phys. 139, 164101 (2013).
